# The “state of the art” of intraoperative neurophysiological monitoring: An Italian neurosurgical survey

**DOI:** 10.1016/j.bas.2024.102796

**Published:** 2024-04-16

**Authors:** Riccardo Antonio Ricciuti, Fabrizio Mancini, Giusy Guzzi, Daniele Marruzzo, Alessandro Dario, Alessandro Della Puppa, Alessandro Ricci, Andrea Barbanera, Andrea Talacchi, Andreas Schwarz, Antonino Germanò, Antonino Raco, Antonio Colamaria, Antonio Santoro, Riccardo Boccaletti, Carlo Conti, Carlo Conti, Nunzia Cenci, Christian Cossandi, Claudio Bernucci, Corrado Lucantoni, Giovanni Battista Costella, Diego Garbossa, Donato Carlo Zotta, Federico De Gonda, Felice Esposito, Flavio Giordano, Giancarlo D'Andrea, Gianluca Piatelli, Gianluigi Zona, Giannantonio Spena, Giovanni Tringali, Giuseppe Barbagallo, Carlo Giussani, Maurizio Gladi, Andrea Landi, Angelo Lavano, Letterio Morabito, Luciano Mastronardi, Marco Locatelli, Michele D'Agruma, Michele Maria Lanotte, Nicola Montano, Orazio Santo Santonocito, Angelo Pompucci, Raffaele de Falco, Franco Randi, Sara Bruscella, Ivana Sartori, Francesco Signorelli, Luigino Tosatto, Roberto Trignani, Vincenzo Esposito, Gualtiero Innocenzi, Sergio Paolini, Vincenzo Vitiello, Michele Alessandro Cavallo, Francesco Sala

**Affiliations:** a Neurosurgery, Ospedale Belcolle, Viterbo, Italy; b Neurosurgery, Azienda Ospedaliera di Perugia, Perugia, Italy; c Neurosurgery, ASST Settelaghi, Varese, Italy; d Clinic of Neurosurgery, AOU Careggi, Firenze, Italy; e Neurosurgery, Ospedale Civile San Salvatore, L'Aquila, Italy; f Department of Neurosurgery, AON SS. Antonio e Biagio e Cesare Arrigo H, Alessandria, Italy; g Unit of Neurosurgery, AO San Giovanni Addolorata, Roma, Italy; h Unit of Neurosurgery, Azienda Sanitaria Alto Adige, Italy; i Unit of Neurosurgery, AOU Policlinico G. Martino di Messina, Italy; j Neurosurgery Clinic, Azienda Ospedaliera Sant’Andrea, Roma, Italy; k Unit of Neurosurgery, Azienda Ospedaliera Policlinico Riuniti Foggia, Foggia, Italy; l Neurosurgery Clinic, Azienda Ospedaliera Universitaria, La Sapienza Policlinico Umberto I° Roma, Roma, Italy; m Unit of Neurosurgery, AOU Sassari, Sassari, Italy; n Unit of Neurosurgery, Azienda Ospedaliera S. Maria, Terni, Italy; o Unit of Neurosurgery, ARNAS G.Brotzu, Cagliari, Italy; p Unit of Neurosurgery, AOU Maggiore Della Carità di Novara, Novara, Italy; q Unit of Neurosurgery, ASST Papa Giovanni XXIII, Bergamo, Italy; r Unit of Neurosurgery, Ospedale “Mazzini” ASL Teramo, Italy; s Unit of Neurosurgery, Ospedale Civile SS Annunziata, Taranto, Italy; t Neurosurgery Clinic, AOU Città Della Salute e Della Scienza di Torino, Italy; u Neurosurgery, Ospedale Civile di Pescara, Italy; v Neurosurgery, ASST Valtellina e Alto Lario, Sondrio, Italy; w Neurosurgery Clinic, A.O.U. Policlinico Federico II - Università Degli Studi di Napoli, Italy; x Unit of Pediatric Neurosurgery, Meyer Children's Hospital IRCCS, Firenze, Italy; y University of Florence, Italy; z Neurosurgery, Ospedale Spaziani Frosinone, Italy; aa Neurosurgery, IRCCS Gaslini, Genova, Italy; ab Neurosurgery Clinic, IRCCS Policlinico San Martino, Genova, Italy; ac Neurosurgery, Fondazione IRCCS Policlinico San Matteo, Pavia, Italy; ad Neurosurgery, ARNAS Civico Palermo, Italy; ae Neurosurgery Clinics, Policlinico “G. Rodolico- San Marco”, Catania, Italy; af Neurosurgery Clinic, IRCCS Fondazione Ospedale San Gerardo Dei Tintori di Monza, Università Bicocca, Milano, Italy; ag Neurosurgery Clinic, Azienda Ospedaliero-Universitaria, Ospedali Riuniti di Ancona, Italy; ah Neurosurgery Clinic, Azienda Ospedaliera Universitaria di Padova, Italy; ai Neurosurgery, AOU Ospedaliero Mater Domini di Catanzaro, Italy; aj Neurosurgery, AO Marche Nord, Pesaro, Italy; ak Neurosurgery, Ospedale San Filippo Neri, Roma, Italy; al Neurosurgery Clinic, Fondazione IRCCS Ospedale Maggiore Policlinico di Milano, Università Degli Studi di Milano, Italy; am Neurosurgery, ASO Santa Croce e Carle, Cuneo, Italy; an Unit of Functional Neurosurgery, AOU Città Della Salute e Della Scienza di Torino, Italy; ao Neurosurgery Clinic, Fondazione Policlinico Universitario Agostino Gemelli IRCCS, Roma, Italy; ap Neurosurgery, Azienda USl Toscana Nordovest, Ospedale di Livorno, Italy; aq Neurosurgery, Ospedale Santa Maria Goretti, Latina, Italy; ar Neurosurgery, Ospedale Santa Maria Delle Grazie di Pozzuoli, Napoli, Italy; as Neurosurgery, Ospedale Pediatrico Bambino Gesù, Roma, Italy; at Neurosurgery, AORN Sant'Anna e San Sebastiano, Caserta, Italy; au Unit of Epilepsy Neurosurgery, ASST GOM Niguarda, Milano, Italy; av Clinic of Neurosurgery, Policlinico Universitario Bari, Bari, Italy; aw Neurosurgery, Ospedale Bufalini Cesena, Italy; ax Neurosurgery, Ospedali Riuniti di Ancona, Italy; ay Neurosurgery, IRCCS Neuromed - Pozzilli, Isernia, Italy; az Neurosurgery, Ospedale San Giovanni Bosco Napoli, Italy; ba Neurosurgery, Ospedale Sant’Anna, Ferrara, Italy; bb Neurosurgery Clinic, Azienda Ospedaliera Universitaria di Verona, Verona, Italy

**Keywords:** IOM, Awake surgery, Eloquent areas, PEM, PES, Brain tumors

## Abstract

**Introduction:**

Intraoperative Neurophysiological Monitoring (IOM) is widely used in neurosurgery but specific guidelines are lacking. Therefore, we can assume differences in IOM application between Neurosurgical centers.

**Research question:**

The section of Functional Neurosurgery of the Italian Society of Neurosurgery realized a survey aiming to obtain general data on the current practice of IOM in Italy.

**Materials and methods:**

A 22-item questionnaire was designed focusing on: volume procedures, indications, awake surgery, experience, organization and equipe. The questionnaire has been sent to Italian Neurosurgery centers.

**Results:**

A total of 54 centers completed the survey. The annual volume of surgeries range from 300 to 2000, and IOM is used in 10–20% of the procedures. In 46% of the cases is a neurologist or a neurophysiologist who performs IOM. For supra-tentorial pathology, almost all perform MEPs (94%) SSEPs (89%), direct cortical stimulation (85%). All centers perform IOM in spinal surgery and 95% in posterior fossa surgery. Among the 50% that perform peripheral nerve surgery, all use IOM. Awake surgery is performed by 70% of centers. The neurosurgeon is the only responsible for IOM in 35% of centers. In 83% of cases IOM implementation is adequate to the request.

**Discussion and conclusions:**

The Italian Neurosurgical centers perform IOM with high level of specialization, but differences exist in organization, techniques, and expertise. Our survey provides a snapshot of the state of the art in Italy and it could be a starting point to implement a consensus on the practice of IOM.

## Highlights

• Despite the diffusion of IOM in the neurosurgical practice, no specific neurosurgical guidelines exist and the complexity of IOM may lead to significant differences across centers.
• In Italy, the vast majority of Neurosurgical centers perform IOM with high level of specialization and in 83% of cases, neurosurgeons describe the IOM implementation by their Institution as satisfying.
• Even centers that perform high volume of IOM express the need for a better internal organization and for an improvement of team coordination, in particular with neurophysiologists.
• Trust in the value of IOM seems to increase with its use and experience. This may reflect the fact that as neurosurgeon became more familiar with IOM, they can better appreciate the value of the different IOM techniques.
• The survey provides a snapshot of the state of the art in Italy and it could be a starting point to address future strategies to implement a consensus on the practice of IOM.

## Graphical abstract

**Figure:**
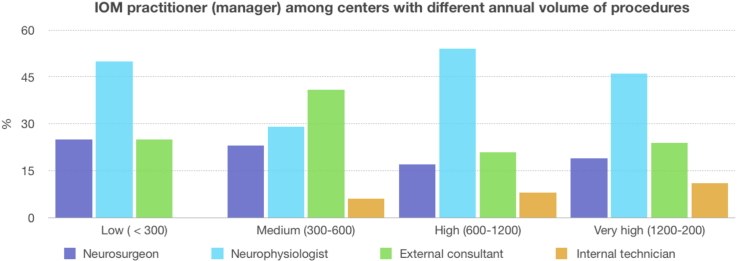

## 1 Introduction

Intraoperative neurophysiological monitoring (IOM) encompass a set of different techniques for neurophysiological real-time monitoring, widely used in Neurosurgery for both, cranial and spinal procedures (Gonzalez et al., 2009; Sala et al., 2002; Kombos et al., 2009; Duffau, 2005; Wong et al., 2022). The aim of IOM is reducing the risk of neurological injury by providing the neurosurgeon with a feedback about his/her action on anatomical structures, so that the procedure can be oriented and guided (Obermueller et al., 2015; Penfield and Gage). Despite IOM is a well-established discipline in neurosurgery, there are important differences in its use between Neurosurgical centers. While the aim of IOM is to minimize the risk of intraoperative neurological injury, some neurosurgeons are not yet confident with these techniques and others argue that, in case of false-positive neurophysiological feedback, IOM may negatively impact on the extent of tumor resection (Bejjani et al., 1998; Fisher et al., 1995; Wiedemayer et al., 2002). Thus, while some neurosurgeons would not perform some procedures without IOM, others may feel IOM as a limitation to their surgical action (Cabraja et al., 2009). The reliability of IOM is thus fundamental to gain the trust of neurosurgeons, but data regarding correlations between IOM and outcome, although available, are heterogeneous and difficult to compare. The differences between centers in the use of IOM may explain the lack of homogeneous data. Indeed, there is no clear indication also regarding which types of surgical procedures should undergo IOM. While, for example, it is almost always used for intramedullary spinal cord tumors (Sala et al., 2022; Ghadirpour et al., 2018; Scibilia et al., 2016), some neurosurgeons do not use IOM in brain tumor surgery (Cabraja et al., 2009). As a whole, IOM includes many different neurophysiological methods, complementary and partially overlapping in the information they provide, each technique supported by different tools, device and procedures. The composition of the IOM team in the operating room is also highly variable and not clearly defined, depending on the medical background, type and duration of the IOM training, personal experience. Equipment and case load may also vary substantially.

Despite useful recommendations on different aspect of IOM already exist (Gertsch et al., 2019; MacDonald et al., 2013, 2019), specific guidelines are still lacking in many areas of IOM.

In 2015 a joint document of the Italian Society of Neurosurgery and the Working Group on IOM of the Italian Society of Clinical Neurophysiology promulgated some IOM recommendations. Seven years later the section of Functional Neurosurgery of the Italian Society of Neurosurgery (SINch) planned and realized this Italian survey on IOM, aiming to obtain general data on the current practice of IOM in Italy.

## 2 MATHERIALS and methos

This survey focused on different aspects of the use of IOM in Italian Neurosurgical academic and non-academic centers. A 22-item, closed-ended, questionnaire (Appendix 1) was built taking into account, among others, these main variables: volume of IOM procedures per year, types of surgical procedures monitored (supratentorial, infratentorial, spinal surgery, peripheral nerves surgery), IOM in awake surgery, experience and know-how of the centers, IOM organization and composition of the IOM team. The survey was sent by email to all Italian Neurosurgery centers and data were collected from May to September 2022.

## 3 Results

A total of 122 Italian Neurosurgical centers were enrolled in the survey; among these, 54 completed the survey questionnaire, covering all geographical areas (north = 15, center = 26, south and islands = 13). All 54 Neurosurgical centers performed IOM. The annual volume of neurosurgical procedures varied substantially ranging from low volume (<300 per year) to very high volume (1200–2000 per year) (Table 1); the majority of centers (44%, n = 24) were high volume (600–1200 per year). Regardless of the annual volume of surgeries, IOM was used in roughly 10–20% of the procedures. As expected, only centers with high and very high surgical volume (25 and 64%, respectively) performed >200 IOM procedures per year, while the majority of the centers performed between 50 and 100 IOM procedures per year (43%, n = 23/54). Medium volume centers ranged from 10 to 100 IOM procedures per year. It should be noted that also centers with low surgical volume perform IOM with not negligible frequency (between 10 and 50 IOM procedures per year in 75% of the cases).

**Table 1:** Relationship between annual surgical volume and IOM variables: cases/year, personnel, experience, provider.

	Low < **300 n** = **4**	Medium **300**–**600 n** = **17**	High **600**–**1200 n** = **24**	Very high **1200**–**2000 n** = **9**	**Total n** = **54**
IOM case/year					
<10	1 (25%)				
10–50	3 (75%)	9 (53%)	6 (25%)		
50–100		8 (47%)	12 (50%)	3 (33%)	23 (43%)
>200			6 (25%)	6 (64%)	12 (22%)
**IOM provider**					
Neurosurgeon	1 (25%)	4 (23%)	4 (17%)	1 (11%)	10 (19%)
Neurologist/clinical neurophysiologist	2 (50%)	5 (29%)	13 (54%)	5 (55%)	25 (46%)
External consultant	1 (25%	7 (41%)	5 (21%)		13 (24%)
Technician		1 (6%)	2 (8%)	3 (33%)	6 (11%)
**IOM experience (in year)**					
<2	1 (25%)	2 (12%)			3 (5%)
2–5		7 (41%)	10 (42%)	2 (22%)	19 (35%)
5–10	3 (75%)	5 (29%)	6 (25%)	1 (11%)	15 (28%)
>10		3 (18%)	8 (33%)	6 (67%)	17 (31%)
**IOM experience (in number of procedure)**					
<50	2 (50%)	2 (12%)			
50–100		4 (24%)	8 (33%)		
100–300	1 (25%)	5 (29%)	5 (21%)	1 (11%)	
300–500		2 (12%)	2 (8%)	1 (11%)	
>500	1 (25%)	4 (24%)	9 (37%)	7 (78%)	
**IOM availability**					
always on request	3 (75%)	10 (59%)	21 (87%)	8 (89%)	
limited	1 (25%)	7 (41%)	3 (13%)	1 (11%)	

### 3.1 IOM experience and institutional organization

The practitioner who performs IOM may vary between Institutions. Globally, in 46% of the cases (n = 25) is either a neurologist or a clinical neurophysiologist, followed by an external consultant typically a IOM technician (24%, n = 13), neurosurgeon (19%, n = 10) and in-house technician (11%, n = 6). When external consultants or technicians were the only IOM professional in the O.R., it was specified that the neurosurgeon provided supervision and assumed medical liability. The use of external IOM consultants (generally a technician working for a private IOM company supplying both the equipment and the personnel) is higher for medium volume centers (41%, n = 7/17) than for very high volume centers, which predominantly relied on internal resources. Regardless of the professional profile, years of IOM experience are heterogeneous: 2–5 year (35%, n = 19), 5–10 year (28%, n = 15) and >10 year (31%, n = 17). It should be noted that in centers with very high volume the practitioner has more than 10 years of experience in 66% (n = 6) of cases, while rarely falls under 2 years (only for low and medium volume centers, in 25% and 12% of cases respectively).

### 3.2 Supratentorial pathology

Almost all centers use IOM for intra-axial tumors (98%, n = 53), while for functional neurosurgical procedures, extra-axial tumors and vascular disease, IOM has been used in less than half of centers (44% n = 24, 39% n = 21, 39% n = 21 respectively, Table 2). Almost all Neurosurgical centers perform MEPs (94%, n = 51), SSEPs (89%, n = 48), direct cortical stimulation (85%, N = 46); the majority of centers also performs subcortical stimulation (80%, n = 43), phase reversal (70%, n = 38) and electrocorticography (61%, n = 33). When direct cortical stimulation (DCS) is used (n = 46), it is only monopolar in 13% (n = 6), only bipolar in 13% (n = 6) both in 74% (n = 34); when subcortical stimulation (SCS) is used (n = 43), it is only monopolar in 5% (n = 5%), only bipolar in 35% (n = 15) both in 60% (n = 26). Other IOM techniques anecdotally used include D wave (5%, n = 3), EMG (4%, n = 2), SCS with ultrasonic surgical aspirator, oculomotors nerves monitoring, cortico-cortical evoked potentials (CCEPs) and deep brain stimulation (DBS) (all at 2%, n = 1).

**Table 2:** Supratentorial pathology: indications and technique.

	n = 54
**Indication**	
functional	24 (44%)
intra-axial tumors	53 (98%)
extra-axial tumors	21 (39%)
vascular	21 (39%)
**Techniques**	
MEPs	51 (94%)
SSEPs	48 (89%)
Direct Cortical stimulation (DCS)	46 (85%)
Subcortical stimulation (SCS)	43 (80%)
Phase reversal	38 (70%)
Electrocorticography (ECoG)	33 (61%)
D wave	3 (5%)
EMG	2 (4%)
SCS with ultrasonic surgical aspirator	1 (2%)
Oculomotor nerves	1 (2%)
Cortico-cortical evoked potential (CCEP)	1 (2%)
Deep brain stimulation (DBS)	1 (2%)

### 3.3 Posterior fossa surgery

IOM is performed in posterior fossa surgery (Table 3) in 95% (n = 51) of centers and, among these, the main pathologies monitored include: extra-axial tumors (88%, n = 45), intra-axial tumors (80%, n = 41), neurovascular conflicts (57%, n = 29), vascular diseases (31%, n = 16) and Chiari malformations (25%, n = 13). Neurovascular conflicts involve the following cranial nerve: VII (100%, n = 29), V (92%, n = 27) and IX (54%, n = 17).

**Table 3:** Infratentorial pathology: indications and technique.

	n = 51
Indication	
extra-axial tumors	88% (45)
intra-axial tumors	80% (41)
neurovascular conflict	57% (29)
vascular disease	31% (16)
Chiari malformation	25 (13)
**Techniques**	
SSEPs	43 (84%)
Cortico-spinal MEPs	43 (84%)
Cranial nerve with free-running EMG	46 (90%)
Cranial nerve with cortico-bulbar MEP	34 (67%)
BEARs	35 (69%)
Blink reflex	25 (49%)

### 3.4 Spinal surgery

All centers included in the survey perform IOM during spinal surgery (Table 4). The IOM is used in the following types of surgery: spinal tumors 98% (n = 53), vascular pathologies 68% (n = 37), instrumented degenerative pathology 52% (n = 28) and non-instrumented degenerative pathology 17% (n = 9). Among centers that use IOM for spinal tumor surgery, the site of the lesions was: intramedullary 96% (n = 51), intradural extramedullary 83% (n = 44) and extradural 45% (n = 24). All centers (100%, n = 54) use PEM and PES; other types of IOM used in spinal surgery included EMG/ENG in 80% (n = 43), D-wave in 74% (n = 40) and dorsal column mapping in 24% (n = 13).

**Table 4:** Spinal surgery: indication and technique.

	n = 54
Surgical pathology	
tumors	53 (98%)
vascular	37 (63%)
degenerative instrumented	28 (52%)
degenerative non instrumented	9 (17%)
**Types of tumors**	
intramedullary	51 (96%)
intradural extramedullary	44 (83%)
extradural	24 (45%)
**Technique**	
MEP	54 (100%)
SEP	54 (100%)
EMG/ENG	43 (80%)
D-wave	40 (74%)
dorsal column mapping	13 (24%)

### 3.5 Peripheral nerves surgery

A total of 27 centers (50%) perform IOM during peripheral nerves surgery. All centers use IOM for tumors surgery and 4 of these also during reconstructive surgery. All centers use direct stimulation of the nerves and almost all (93%, n = 25) also use EMG/ENG.

### 3.6 Awake surgery

Awake surgery is performed by 70% (n = 38) of centers (Table 5), all for intra-axial tumors (100%), but also for functional neurosurgery (34%, n = 13), extra-axial tumors (24%, n = 9) and vascular surgery (5%, n = 13). Both hemispheres are monitored in 66% (n = 25) of cases. In the majority of centers (n = 25, 66%) not only cognitive functions and speech are monitored, but also other brain functions visual function, neglect and calculation. Techniques include: MEPs and SEPs, DCS, SCS, visual evoked potentials, EcoG and free-run electromyography. A multidisciplinary team is involved and the three main figures are: dedicated neurosurgeon (n = 32), neuropsychologist (n = 34) and neuroanesthesiologist (n = 28).

**Table 5:** IOM and awake surgery: indication, strategies and personnel.

	n = 38
Indication	
Intra-axial tumors	38 (100%)
Functional	13 (34%)
Extra-axial tumors	9 (24%)
Vascular	5 (13%)
**Strategies**	
Only dominant hemisphere monitoring	13 (34%)
Both	25 (66%)
Only cognitive function (including speech)	13 (34%)
Also other brain functions	25 (66%)
**For tumors in motor cortex**	
Sometimes	10 (26%)
Always	20 (53%)
No	8 (21%)
**Team**	
Dedicated surgeon	32 (84%)
Neurophysiologist (from unit)	20 (53%)
Neurophysiologist (external)	5 (13%)
Technician (from unit)	23 (61%)
Technician in service	13 (34%)
Speech therapist	11 (29%)
Neuroanestesiologist	28 (74%)
Neuropsychologist	34 (89%)

### 3.7 Who performs IOM in the operating room?

Regarding the organization of IOM in the operating room, Neurosurgical centers may be classified in one of these categories: the doctor responsible for IOM and the technician are both always present in the operating room during the procedure (43%, n = 23); the technician is always present in operating room during the procedure, while the doctor responsible for IOM is present in the critical phase of the surgery or whenever called by the neurosurgeon and/or by the technician (22%, n = 12); the technician is always present in operating room during the procedure, there is no other medical doctor and the neurosurgeon is responsible for IOM (35%, n = 19).

### 3.8 Satisfaction about IOM implementation by institution

Globally, in 83% of cases (n = 45), the surgeon refers that IOM implementation is adequate to the request. Among these, in 47% (n = 21) the service is guaranteed by external resources (technician, specialist) while in 53% (n = 24) the centers relies on internal human resources only. In 17% of cases (n = 9) IOM resources have been judged as inadequate to the request.

## 4 Discussion

We presented a survey on the use of IOM in Neurosurgery that involved a total of 54 Italian Neurosurgical centers, aiming to obtain a snapshot of the state of the art of neuromonitoring in Italy. We conceived the survey believing that despite the widespread use of IOM and given its complexity, a high level of heterogeneity among different centers and surgeons may exist.

About 50% of Italian Neurosurgical centers participated to the survey, including most of the academic institutions, and 100% of these use IOM, including centers with low-volume of annual surgical procedures. We therefore cannot exclude a selection bias as it is possible that centers were IOM is not or only seldom used tended not to participate in the survey. If so, this survey may not completely reflect the “average” IOM practice in Italian Neurosurgical centers.

The survey has included centers with different volumes of surgical procedures per year, from less than 300 to more than 2000 operations per year. Overall, IOM is widely used with a mean of 10–20% of neurosurgical procedures performed under neurophysiological guidance. Even 75% of low-volume neurosurgical centers perform up to 50 IOM procedures per year, while high and very high-volume centers use IOM in more than 200 cases per year. The fact that the percentage of monitored procedures is between 10% and 20% independently of the total surgical volume, may suggest uniformity in the IOM indications with regards to specific pathologies and surgeries.

Despite the diffusion of IOM in the neurosurgical practice, no specific neurosurgical guidelines exist and the complexity of IOM may lead to significant differences across centers. Recommendations regarding various aspects of IOM, such indications (MacDonald et al., 2013, 2019; Martin and Stecker, 2008), team composition and technical instrumentation (Gertsch et al., 2019; American Association of Electrodiagnostic Medicine, 1999), have been published, but it should be noted that these do not represent an obligatory standard for IOM.

The lack of consensus in IOM practice may be due to various aspect. First, at the beginning of IOM using, some neurosurgeon they did not trust very much in IOM alarm and they prefer anatomical feedback in guiding the surgery. This issue has now been overcome, since there is increasing use of IOM in neurosurgical practice and the specificity and sensitivity of the technique are been widely confirm even by accumulating data and personal experience. A major limit to gain consensus and guideline is now the different organization between center, regarding technique, procedures and team composition. This is why we focused our survey mainly on practical aspect of IOM, aiming to obtain general data and detect the heterogeneity of IOM practice in centers with different experience and volume of procedures.

The survey suggest that most centers perform IOM with a consolidated experience. In only 5% of centers the professional responsible for IOM had less than 2 years of IOM experience. As expected, this experience is related to the surgical volume, but not in a linear fashion: for example, 25% of low-volume centers has an experience of more than 500 IOM procedures, that is the volume which is prevalently reported by very-high volume centers.

As suggested by the international survey by Cabraja et al. (2009), trust in IOM is not dependent on the general experience of the neurosurgeon, but rather on the specific experience with IOM and correlates with the number of monitored procedures. This influences also the way in which IOM is used: while surgeon with less IOM experience are more influenced by IOM data during surgery, the ones with more surgical experience are less prone to change the surgical strategy based on IOM data only, and this could be interpreted as a more critical use of IOM by more experienced neurosurgeons.

Expert surgeon may use IOM in a more “targeted” way, since they are able to put the IOM feedback in the surgical context. Thus, they may change the surgical strategy based on IOM alarm less frequently compared to non expert surgeon. This does not mean less trust in IOM by expert surgeon, rather a more critical and specific use of IOM.

The ultimate role of IOM is to tailor surgical maneuvers based on neurophysiological real time data. Yet, unlike the anatomical data which is immediately and directly available to the surgeon, IOM data are mediated by the recording and interpretation of the neuromonitoring professional, thus needing a strict cooperation and communication between the two as well as with the anesthesiologist. There are differences in who performs IOM in different centers. Independently of the annual volume of surgical procedures, the neurosurgeon is the IOM expert in 19% of centers while in 46% of the centers is either the neurologist or the neurophysiologist responsible for the IOM activity in the operating room. In the remaining cases, the IOM-expert is a technician or external consultant.

This heterogeneity in IOM organization in operating room may explain one of the issue which emerge from the survey, regarding the need for a strict coordination among the IOM team, in particular with neurophysiologist. The availability of technician or neurophysiologist, for example in urgent surgical procedure, is not the routine, even in centers with very high volume of IOM procedures.

One of the controversial aspects of IOM is related to the definition of “neuromonitoring expert” (American Association of Electrodiagnostic Medicine, 1999). What should be the credentials for independent, unsupervised, IOM activity in the operating room? Can a neurosurgeon supervise and be medically liable for IOM if he/she has no proven expertise in IOM? What about a clinical neurophysiologist who has never practiced in the operating room? This is a potential issue and leads to the question whether a IOM certification, independently of medical background, should be introduced. While in low- and medium volume centers neurosurgeon is often the main IOM expert (50% and 70% respectively), neurologist or neurophysiologist are more often involved in high- and very-high volume centers. This can be interpreted as if the IOM background and experience of the neurosurgeon is more important in low-volume centers.

Intra-axial brain tumors are the main pathology for which IOM is used (about 98% of centers). The main IOM techniques reported are MEPs (94%), SSEPs (89%), DCS (85%) and subcortical stimulation (80%). Intraoperative MEPs are the gold standard for supratentorial glioma surgery in eloquent areas (Al-Adli et al., 2023). The two techniques by which the MEPs can be evoked are transcranial stimulation (TES), that utilize a high-voltage electrical stimulus through the scalp/skull to activate the motor cortex and descending pathways, and DCS, by stimulating through a strip electrode that is placed over the primary motor cortex (Abboud et al., 2021). The predictive value for postoperative motor deficit is high, but it has been suggested that DCS has a higher reliability, independently of the location of the tumors and the position of the patient (Viganò et al., 2022). Direct cortical stimulation is often used in combination with electrocorticography (ECoG), which ensures a bidirectional flow of information, stimulation and recording of cortex discharge, increasing the safety of stimulation (Caldwell et al., 2019; Merton and Morton, 1980) In our survey ECoG is used by 61% of centers and subcortical stimulation (SCS) in 80%. Subcortical stimulation aims to identify the white matter functional boundaries, which represent the limits for the resection (Deletis and Sala, 2011; Han et al., 2018). With regards to the corticospinal tract, when this can be identified during stimulation mapping, the distance of the tracts from the stimulating probe is approximately based on a one to one correlation: each mA of amplitude corresponds to about 1 mm of distance from the tract. Consistent data in the literature suggests that a threshold of 3 mA or lower is associated to a significantly higher risk of postoperative motor deficit. A 7.6% rate of permanent motor deficit has been reported with positive subcortical mapping as compared to only 2.3% in the case in which the tract could not be identified (Keles et al., 2004). Despite somatosensory mapping (SSEPs) is inferior to MEPs in its predictive value for post-operative motor deficit (Thirumala et al., 2013; Rossi et al., 2021; Wiedemayer et al., 2004), in our survey SSEPs are still largely used during brain tumor surgery. One of the main advantages of SSEPs is the identification of the central sulcus by the phase reversal technique (Cedzich et al., 1996), that is used in 70% of centers.

For vascular neurosurgery, aneurism clipping in particular, IOM is widely employed (Park et al., 2021), with a percentage of 39% in our survey. During aneurysm clipping the rate of postoperative ischemia has been reported in the range of 6–14% without IOM (Alshekhlee et al., 2010) as compared to 1–8% when neuromonitoring was used (Nasi et al., 2020; Chung et al., 2018).

The role of IOM for meningiomas in the rolandic region is less defined (Paldor et al., 2022; Maiuri and Corvino, 2023), but still 39% of the centers reported its use, reflecting an increasing rate of indications for the use of IOM in the daily neurosurgical practice.

Awake craniotomy with IOM is considered the standard of care for resection of tumors in eloquent areas (Duffau, 2018; , Hervey-Jumper et al., 2015; Kim et al., 2009). It allows accurate identification and preservation of critical cortical areas and subcortical pathways. The majority of centers (70%) perform awake surgery, all for intra-axial tumors. The protocol generally adopted (asleep-awake-asleep) provides the interaction with the patient only during cortical and subcortical mapping with direct electrical stimulation (DES). The functions most often tested during awake craniotomies are usually language, motor, somatosensory, visual and visuospatial (Saito et al., 2018). In our survey, both hemispheres are generally tested and 66% of centers extend the IOM beyond language and motor function. For tumors in motor areas, half of the centers always chose awake craniotomy with IOM as standard surgery. The largest European survey focusing on treatment strategy for tumor in eloquent areas, including the use of IOM (Spena et al., 2017), analyzed a total of 2098 patients, showing that in 41% of the cases neurosurgeons preferred awake surgery with IOM while in 58.8% of cases they preferred asleep surgery with IOM and mapping. The specific advantage of awake craniotomy is the possibility of continuous assessment of motor function during tumor resection, extended not merely to the voluntary movement but also to sensori-motor integration, allowing early recognition of deterioration and optimization of surgical strategy (Chacko et al., 2013).

Posterior fossa surgery is performed by 95% of centers and IOM is used for both extra-axial (88%) and intra-axial tumors (80%). Studies regarding IOM in posterior fossa surgery are commonly focused on cranial nerve (CN) monitoring (Broggi et al., 1995) while the value of MEPs and SEPs has been less investigated, although a multimodality approach is advocated (Slotty et al., 2017; Cheek, 1993; Sala et al., 2015). The variety of techniques reported in the survey includes: MEPs, SEPs, free-run EMG, cortico-bulbar motor evoked potential (C-MEP), auditory evoked potentials (BAERs) and Blink-reflex.

Spinal surgery is the field of neurosurgery in which IOM is mostly applied, with 100% of centers using IOM for spinal procedures. Although IOM cannot always prevent intraoperative injury, in most cases it can at least mitigate the damage. A large multi-center study on surgery for spinal deformity showed a reduction of 50% on postoperative neurological deficit in monitored procedures compared to non-monitored, with a percentage of deficit with constant stable SSEPs that was only 0.063% (Nuwer et al., 1995; Biscevic et al., 2020). Despite the growing credit given by spinal surgeons to IOM, no established consensus exists and its use shows high variability between centers (Lall et al., 2012; Drake et al., 2010). Moreover, some systematic reviews showed low level of evidence that IOM reduce the rate of new neurologic deficit and very low evidence about the efficacy of intraoperative alert on preventing injury (Fehlings et al., 2010). It should be yet emphasized that spine and spinal cord surgery includes a variety of surgical procedures with very different risk for neurological injury and, accordingly, the indications for the use of IOM, and its value, vary significantly.

Spinal tumors may be classified as extra-dural, intra-dural extramedullary and intramedullary and the use of IOM in our survey based on anatomical location of the tumor is 45%, 83% and 96% respectively. The combination of MEP and SSEPs is considered the gold standard for spinal surgery monitoring (Charalampidis et al., 2020; Nattawut and Peeranut, 2023), with sensitivity and specificity approaching 100%. As expected, all centers report the use of both MEP and SSEPs. In the case of intramedullary spinal cord tumor surgery, data showed that the addition of D-wave, which is used by 74% of the centers in our survey, may increase the prediction of long-term motor function (Kothbauer et al., 1998).

Use of IOM for lumbar surgery, such as uncomplicated laminectomy and microdiscectomy, is more controversial. This emerge also from this survey, in which the percentage of centers using IOM for instrumented and non-instrumented degenerative spine pathology dropped to 52% and 17% respectively. Although many surgeon are confident with IOM and suggest that monitoring in all cases is beneficial, it is not clear whether IOM affected the already low neurological complication rates. In instrumented spine surgery or revision surgery, where there is the risk of nerve injury, spontaneous (or free-running) and triggered EMG can improve detection of nerve root injuries and are used by 80% of centers.

## 5 Limitations and conclusions

This survey was aimed to reflect the current practice of IOM in Italian Neurosurgical centers. Although many institutions participated in the survey, representing most of the largest and academic centers, 56% of the centers did not reply. It may well be that monitoring is not used or used to a lesser extent in centers that did not participate in the survey. With this in mind, still we consider the survey quite representative of the current trends in IOM in Italy. These appear in line with the existing literature. The vast majority of the centers perform IOM, even with high level of specialization, but differences exist in the organization of the procedures, techniques, indications and expertise of the IOM team. It is interesting that in 83% of cases, neurosurgeons describe the IOM implementation by their Institution as satisfying, regardless whether IOM was provided by an in-house team or outsourced to an external provider. On the open-ended questions, three main topics arose. First, IOM is considered as indispensable in modern neurosurgical practice, and the trust in its value seems to increase with its use and experience. This may reflect the fact that as neurosurgeon became more familiar with IOM, they can better appreciate the value of the different IOM techniques. This may open a discussion on the value to include basic IOM knowledge in the neurosurgical curriculum.

Second, even centers that perform high volume of IOM express the need for a better internal organization and for an improvement of team coordination, in particular with neurophysiologists. Third, in spite of the increasing use of IOM by neurosurgeons, there is still a lack of consensus on credentials, indications and techniques. In this regard, as much as this survey focused on general aspects of IOM practice, it provides a snapshot of the state of the art in Italy and it could be a starting point to address future strategies to implement a consensus on the practice of IOM.

## Declaration of competing interest

The authors declare that they have no known competing financial interests or personal relationships that could have appeared to influence the work reported in this paper.
