# Supplementary material for: The “state of the art” of intraoperative neurophysiological monitoring: An Italian neurosurgical survey
Source: Brain Spine. 2024 Apr 16;4:102796. doi: 10.1016/j.bas.2024.102796 (PMC11063224; doi:10.1016/j.bas.2024.102796)
Supplement: Multimedia component 1 [file mmc1.pdf]

IOM SURVEY

Number of surgical procedures per year
<300
300 - 600
600 – 1200
1200 – 2000

Do you use Intraoperative Neuromonitoring (IOM) ?
Yes
No

Availability of IOM in your Neurosurgical center
Yes (always when required)
No
Limited

Number of monitored surgical procedures per year
< 10 per year
10-50/year
50/100/year
>200/year

Who is the IOM manager in your center?
Neurosurgeon
Neurologist / Clinical Neurophysiologist
Technician
External consultant (medical doctor)
External consultant (technician)
Other

How many year has the IOM manager involved in monitoring?
< 2 years
2-5 years
5-10 years
> 10 years

What is the IOM manager level of experience in your center?
< 50 monitored procedures
50-100 monitored procedures
100-300 monitored procedures
300-500 monitored procedures
> 500 monitored procedures

In which anatomical site of pathology do you use IOM?
Supratentorial
Posterior fossa
Spinal
Peripheral nerve

If supratentorial surgery: for which pathologies ?
Intra-axiax tumors
Extra-axia tumors
Vascular
Functional neurosurgery

Which kind of IOM do you use?
MEP
SSEP
ECOG
PhaseReversal
Cortical stimulation
Subcortical stimulation
Other

Do you perform "awake surgery"?
Yes
No

If you perform "awake surgery", in which pathologies?
Intra-axiax tumors
Extra-axia tumors
Vascular
Functional neurosurgery

Do you perform "awake surgery" only for left brain?
Yes
No

Only language and cognitive functions are monitored in "awake surgery"?
Yes
No

Do you perform "awake surgery" for a tumor interesting only motor cortex?
Yes
No
Sometimes

In "awake surgery", your team is composed by:
Dedicated surgeon
Neuropsychologist
Neurophysiologist (internal)
Neurophysiologist (external, other centers)
Technician (internal)
Technician (external, other centers)
Technician in service
Dedicate Neuroanesthesiologist
Logopedist
Other

If posterior fossa surgery: for which pathologies do you use IOM?
Intra-axiax tumors
Extra-axia tumors
Vascular
Neurovascular conflict
Chiari malformation
Other

Which kind of IOM do you use for posterior fossa surgery?
SSEP
MEP
EMG-ENG

Cranial nerves monitoring

If spinal surgery: for which kind of pathologies do you use IOM?
Tumor (intra-dural, extramedullary, intramedullary)
Vascular
Instrumented degenerative (cervical, dorsal, lumbar)
Non-instrumented degenerative

Which kind of IOM do you use in spinal surgery?
SSEP
MEP
D-wave
EMG/ENG

If peripheral nerve surgery: which kind of pathology?
Tumors
Reconstructive surgery

Which kind of IOM do you use in peripheral nerve surgery?
EMG/ENG
Stimulation

How do you define IOM activity in your center:
Appropriate to request, with internal resource
Appropriate to request, but with external resource (consultant, technician, ecc.)
Not appropriate for lack of experience and dedicated staff
Not appropriate for lack of device

Which of the following model better describe the IOM organization in your center:
Medical doctor (neurophysiologist) IOM manager and technician alway present in operating room
Technician always present in operating room and medical doctor IOM manager called only in critical phases (requested by Neurosurgeon)
Technician always present in operating room, neurosurgeon is the IOM manager and no other persons are present
Automatic IOM with no technician in operating room
