# Supplementary material for: The “state of the art” of intraoperative neurophysiological monitoring: An Italian neurosurgical survey
Source: Brain Spine. 2024 Apr 16;4:102796. doi: 10.1016/j.bas.2024.102796 (PMC11063224; doi:10.1016/j.bas.2024.102796)
Supplement: Multimedia component 2 [file mmc2.pdf]

GENTILE COLLEGA,

la Neurochirurgia utilizza sempre più frequentemente ausili nell'intenzione di migliorare l'outcome del paziente rispetto al risultato clinico atteso. I monitoraggi neurofisiologici intraoperatori rappresentano un aiuto tecnico impiegato nella nostra pratica clinica a tal fine.

E' con l'intenzione di fornire alla nostra società scientifica un quadro generale dell'utilizzo dei monitoraggi neurofisiologici intraoperatori nel panorama delle Neurochirurgie Italiane che la Sezione di Neurochirurgia Funzionale della Società Italiana di Neurochirurgia, propone la seguente Survey.

Ti ringraziamo anticipatamente per la Tua preziosa collaborazione.

Struttura Ospedaliero/ Universitaria presso la quale lavora (Facoltativo)………………………………………………………………………………………………

Regione (Obbligatorio)………………………………………………………………………………

- **Numero di interventi effettuati per anno presso la tua Unità Operativa**
    - <300
    - 300 - 600
    - 600 – 1200
    - 1200 – 2000

- **Utilizzi nella tua pratica professionale ilmonitoraggio neurofisiologico intraoperatorio (MNI)?**
    - SI
    - No

- **Disponibilità del MNI presso la vostra struttura ospedaliera:**
    - a) Si (sempre disponibile quando richiesto)
    - b) No
    - c) Disponibilità limitata (se possibile, specificare):…………………………………………………………..

- **Stima del numero di interventi eseguiti con MNI:**
  - < 10/anno
  - 10-50/anno
  - 50/100/anno
  - >200/anno

- **Chi è la figura responsabile del MNI presso il tuo Centro?**
  - Neurochirurgo
  - Neurologo/ Neurofisiologo clinico
  - Tecnico di Neurofisiologia
  - Consulente Esterno (Neurofisiologo o altra figura Medica)
  - Consulente Esterno (Tecnico di Neurofisiopatologia)
  - Altro……………………………………………

- **Da quanti anni il Responsabile del MNI è direttamente coinvolto in questo tipo di attività?**
  - < 2 anni
  - 2 - 5 anni
  - 5 - 10 anni
  - > 10 anni 9.

- **Qual è il grado di esperienza specifica del Responsabile del MNI presso la vostra struttura?**
  - < 50 interventi monitorati
  - 50 - 100 interventi monitorati
  - 100 - 300 interventi monitorati
  - 300 - 500 interventi monitorati
  - > 500 interventi monitorati

- **In quale sedi chirurgiche utilizzi i MNI?**

- Sovratentoriale
- In Fossa Cranica Posteriore
- Nella Chirurgia Spinale/ Midollare
- Nella chirurgia dei NN. Cranici

- **Se Chirurgia Sovratentoriale, in quali patologie utilizzi i MNI?:**
  - Neoplasie intrinseche
  - Tumori Estrinseci
  - Vascolare
  - Funzionale

- **Che tipo di monitoraggi utilizzi?**
  - Potenziali Evocati Somatosensoriali
  - Potenziali Evocati Motori
  - ECOG
  - PhaseReversal
  - Stimolazione Corticale: - Bipolare; - Monopolare; - Entrambe
  - Stimolazione Sottocorticale: - Bipolare; - Monopolare; - Entrambe
  - Altro……………………………………………..

- **Nel Tuo Centro, utilizzi l'Awake Surgery (Chirurgia da Sveglio) come metodica chirurgica?**
  - Si
  - No
- **Se SI, in quali circostanze?**
- Tumori Intrinseci
- Tumori Estrinseci
- Vascolare
- Funzionale
- Altro………………………………………………..

- **Solo nella chirurgia dell'Emisfero Sinistro?:**
  - Si
  - No
- **Monitori solo funzioni cognitive incluso linguaggio?**
  - Si
  - No
  - Altro…………………………………………….
- **Per un paziente con tumore in area motoria non legata al linguaggio, fai awake?**
  - Si
  - No
  - A volte
  - Spiega (Facoltativo)…………………………………………………………………………………….

- **Nell'AWAKE SURGERY, Il Tuo Team è composto:**

Ruolo	Si	No
Chirurgo dedicato		
Neuropsicologo		
Neurofisiologo del Centro		
Neurofisiologo con cui collabori (anche di altro Centro)		
Tecnico di Neurofisiologia del Centro		
Tecnico di Neurofisiologia in Service		
Neuroanestesista dedicato		
Logopedista		
Altro………………………………………..	……………………………………………….	………………………………………………..

- **Se Chirurgia della Fossa Cranica Posteriorein quali patologie utilizzi i MNI?:**
  - Tumori Estrinseci
  - Tumori Intrinseci
  - Conflitti Neurovascolari
    - V N.C.
    - VII N.C.
    - IX N.C.
    - Altro
  - Vascolare
  - Sd. Di Chiari
  - Altro

- **Che tipo di monitoraggi utilizzi?**
  - Potenziali Evocati Somatosensoriali
  - Potenziali Evocati Motori
  - EMG-ENG
  - Monitoraggio NN. Cranici
  - Specificare quali……………………………………………..
  - Altro…………………………………………………………

- **Se Chirurgia Spinale/Midollare:**
  - Tumori
    - Intrarachidei Extra durali
    - Intradurali Extramidollari
    - Intramidollari
  - Vascolare
  - Degenerativa Strumentata
    - Cervicale

- Approcci Anteriori; Specificare……………………………………………….
- Approcci Posteriori; Specificare……………………………………………..

▪ Dorsale
- Approcci Anteriori; Specificare……………………………………………….
- Approcci Posteriori; Specificare……………………………………………….

▪ Lombare
- Approcci Anteriori; Specificare……………………………………………….
- Approcci Laterali; Specificare……………………………………………….
- Approcci Posteriori; Specificare……………………………………………….

○ Degenerativa non strumentata
▪ Se si specificare…………………………………………………………………………….

- Che tipo di monitoraggi utilizzi?
  - Potenziali evocati Somatosensoriali
  - Potenziali evocati Motori
  - Onda D
    - Se Onda D, fino a che livello……………………………………………………..
  - EMG/ ENG

- **Se chirurgia dei Nervi Periferici:**
  - Tumori
  - Ricostruttiva
- Che tipo di monitoraggi utilizzi?
  - EMG/ ENG

○ Stimolazione

- **Ritieni l'attività di MNI presso la vostra struttura:**
  - ○ Adeguata alle esigenze, con risorse interne
  - ○ Adeguata alle esigenze, con risorse esterne (consulente, tecnico di NFP, altro….)
  - ○ Non adeguata per mancanza di personale con le necessarie competenze
  - ○ Non adeguata per la mancanza di attrezzature (apparecchiature IOM) e)
  - ○ Altro: ……………………………………………………………………………..

- **In generale, quale dei seguenti modelli descrive meglio l'attività di MNI presso la vostra struttura:**
  - ○ Il medico responsabile del MNI e il tecnico di NFP sono presenti in sala per tutta la durata dell'intervento b) Il tecnico di NFP è presente in sala per tutta la durata dell'intervento mentre il medico responsabile del MNI è presente in sala per le fasi critiche dell'intervento e/o ogni qualvolta richiesto dal tecnico di NFP o dal neurochirurgo
  - ○ Il tecnico di NFP è presente in sala per tutta la durata dell'intervento. Il neurochirurgo è il medico responsabile del MNI e non vi sono altre figure mediche coinvolte
  - ○ L'attività di MNI è svolta utilizzando sistemi automatici di neuromonitoraggio per cui il responsabile del MNI è il neurochirurgo e non è presente il tecnico di NFP
  - ○ Altro: ……..

- **Commenti** (Max 70 parole)

………
